# Antimicrobial Prescription Habits of Dentists Performing Dental Implant Treatments in Santo Domingo, Dominican Republic

**DOI:** 10.3390/antibiotics9070376

**Published:** 2020-07-03

**Authors:** Juan Manuel Aragoneses, Javier Aragoneses, Vanessa Arlette Brugal, Juan Algar, Ana Suarez

**Affiliations:** 1 Department of Dental Research, Federico Henriquez y Carvajal University, 10106 Santo Domingo, Dominican Republic; juan.aragoneses@ufhec.edu.do; 2 Department of Dentistry, Federico Henriquez y Carvajal University, 10106 Santo Domingo, Dominican Republic; jaragoneses@ufhec.edu.do; 3 Department of Pediatric Dentistry and Orthopedics, School of Dentistry, Universidad Autónoma de Santo Domingo, 10105 Santo Domingo, Dominican Republic; vbrugal99@uasd.edu.do; 4 Department of Clinical Dentistry, School of Biomedical Sciences, Universidad Europea de Madrid, 28670 Madrid, Spain; juan.algar2@universidadeuropea.es; 5 Department of Preclinical Dentistry, School of Biomedical Sciences, Universidad Europea de Madrid, 28670 Madrid, Spain

**Keywords:** antibiotic prophylaxis, prescription behavior, dental implant surgery, Dominican Republic

## Abstract

The use of antibiotics in implant treatments is controversial. The purpose of this research was to study the behaviors of Santo Domingo dentists who prescribe antimicrobials to patients for the placement of dental implants. A total of 99 dentists participated in the study. A share of 1.2% of dentists prescribed antimicrobials solely in the preoperative period, 8.6% after surgery, 44.4% before and after, 19.8% only in specific situations, and 25.9% did not prescribe at all. Amoxicillin was the predominant antimicrobial of choice. A cross-sectional, observational, survey-based study was conducted. The items studied were demographics, self-assessment of knowledge about antibiotics and when they are used, as well as their recommended dosage and duration, in healthy and non-allergic patients. Notable variability was found in the prescription behaviors of antimicrobials. Bridging gaps in knowledge on the subject could help to standardize prescription guidelines.

## 1. Introduction

The rehabilitation of edentulous spaces using implants and their corresponding prosthetic structures is currently considered a routine treatment in dentistry [1]. It is estimated that the global market for dental implants will reach USD $13 billion in 2023 [2], due in part to an aging population with higher chances of suffering tooth loss. 

For more than 30 years, implants have proven to be predictable treatments, with a high long-term success rate (above 90%) [2,3,4]. However, as with any treatment, implants can fail or present complications, placing strain on the patient–dentist relationship [1]. 

Postoperative infections and early implant failures appear to be closely linked to bacterial contamination during surgery [5]. For this reason, it is not uncommon for surgeons to use preoperative antibiotics as a prophylaxis to produce an aseptic environment and thus to reduce potential failure [6,7,8]. In the case of preventive antibiotic coverage, there is no consensus on the use of these drugs in either single or multiple doses, nor has it been established that prescriptions must only be given during the preoperative or postoperative phase [6,9,10]. 

Other studies suggest that antibiotic coverage does not guarantee a drop in potential postsurgical complications [11,12,13,14]. Additionally, there is currently great controversy surrounding the indiscriminate use of antibiotics, as antibiotics have been known to cause adverse effects, ranging from direct organ toxicity (which includes gastrointestinal, hematological, and regular flora alterations leading to opportunistic infections such as *Clostridium difficile*, nephrotoxicity, neuropathies, drug interactions, and hepatobiliary alterations), as well as hypersensitivity reactions that can range from skin reactions to anaphylactic shock [8,10,15]. On the other hand, the risk of these undesirable effects is cumulative and related to increased exposure [16]; this overprescription promote the proliferation of antibiotic-resistant bacteria [10,13,15,17,18]. Moreover, it has been reported that 66% of antibiotics prescribed in connection with dental procedures are not clinically indicated [19] because bacterial cultures and sensitivity tests are rarely performed; therefore, broad-spectrum antimicrobials are prescribed as a result of non-evidence-based assumptions [20]. This proves all the more important when it is estimated that 10% of antimicrobials are prescribed by dentists [21]. Dentists should therefore apply the principles of antibiotic administration and should not use them routinely on healthy patients [22]. 

Since there is no official body in the Dominican Republic in charge of regulating or motivating professionals to follow strict guidelines when making antibiotic prescriptions, and the lack of current consensus among dentists on the use of antibiotics for dental implant surgeries [4,23,24,25,26], this study aims to discover the perceptions of a group of dentists from Santo Domingo (Dominican Republic) regarding their knowledge about antibiotics, the prescription guidelines before and after implant surgeries, as well as post-treatment complications.

## 2. Results

A total of 120 dentists attended the session in question. Of these, 99 completed the surveys. After applying the inclusion and exclusion criteria, a total of 81 surveys were obtained, which, according to a standard probabilistic model with an error α = 5% and a confidence level of 95%, met the minimum permitted size (i.e., 79).

The response rate to the survey was 67.5%. The 81 dentists who participated in the study ranged in age from 28 to 64 years, with an average age of 43.3 years. Regarding sex distribution, more women (63%) than men (37%) took part in the study. This group of professionals had clinical experience that ranged from 5 to 32 years, with a mean of 17.8 years.

When asked to rank their perceived knowledge about antibiotics acquired during their undergraduate studies on a scale of 1–10, the respondents’ mean score was 7.0. Their average perceived knowledge increased (reaching a mean of 7.9) when they confessed to having read scientific literature on the subject and thus acquired greater knowledge. 

Of these 81 professionals, only one dentist prescribed a single antibiotic intake before surgery, constituting 1.2% (*N = 1*) of the sample. A share of 8.6% (*N = 7*) only prescribed antibiotics after surgery. The vast majority, i.e., 44.4% (*N = 36*), prescribed antimicrobials before and after implant placement at all times, while 19.8% (*N = 16*) only prescribed antibiotics before and/or after surgery depending on the situation in question. Finally, 25.9% (*N = 21*) did not routinely prescribe antimicrobials for this procedure (Table 1).

Regarding the choice to prescribe only after surgery, it was observed that the antibiotic of choice was amoxicillin (500 mg) over seven days (*N = 5,* 31.2%), followed by azithromycin (500 mg) over three days (*N = 4,* 25%) (Table 2).

A share of 44% of the participants prescribed antibiotics both before and after surgery (*N = 36*). The antibiotics of choice were amoxicillin (875 mg) and clavulanic acid, prescribed by 52.8% of respondents before surgery and by 61.1% after surgery (*N* = 22), followed by azithromycin (500 mg) with 25% (*N* = 9) before implant placement and 27.8% after surgery (*N* = 10), and then amoxicillin (500 mg) with 13.9% (*N* = 5) before surgery and 11.1% after treatment (*N* = 10) (Table 3).

## 3. Discussion

Dental implants are an increasingly popular treatment option to replace missing teeth. In order to minimize the risk of this undesirable outcome, the use of antimicrobials is a preventive measure worth considering. For this reason, various systematic reviews and meta-analyses have been carried out in recent years to evaluate the efficacy of antibiotics, with varying results. In 2018, Gill et al. [27] concluded that there was no statistical evidence that prescribing antimicrobials as a prophylactic measure reduced the risk of implant failure.

Likewise, Braun et al. [15] stated in 2019 that although antibiotics can reduce implant failure, no definitive conclusions can be made. This statement becomes controversial in the case of healthy patients, due to the inherent risks of treatment with antimicrobials, such as hypersensitivity reactions, direct toxicity, and opportunistic bacterial infections, as well as the proliferation of multidrug-resistant bacteria, which can reduce the effectiveness of the treatment. Conversely, other authors [22,28] have concluded that antimicrobials should not be prescribed as prophylaxis to prevent failures in implantology. Given these findings, and in light of the associated risks of antibiotics for both individual and public health [29], a reassessment of the routine prescription of prophylaxis in dental implant placement procedures is required. Therefore, in the absence of a clear consensus or an established protocol, it can be affirmed that the prescription of antimicrobials to minimize the chances of implant failure continues to be controversial.

This study aimed to shine a light on both the degree of antibiotic knowledge of dentists in Santo Domingo, the Dominican Republic, and the antimicrobial prescription guidelines for implant surgeries being used on the island. It is the first study of its kind with the above characteristics to be carried out in this country.

In the present work, participants performed a self-assessment in which they ranked their knowledge about antimicrobials acquired during their undergraduate studies; an average of 7 out of 10 was obtained. This ranking increased to 8 when it was perceived that their knowledge had been boosted thanks to the reading of scientific literature. Dentists, as health professionals, should be aware of the proper use of drugs. The incorrect use of antibiotics highlights the need to create continuing education courses with accessible formats that are compatible with the dental practice and that will help prevent the unnecessary prescription of antimicrobials [27,30,31]. 

In a 2018 systematic review and meta-analysis by Rodriguez Sánchez et al. [17], the authors concluded that a single dose of amoxicillin preoperatively is efficient and effective in preventing possible implant failures. Romandini et al. [32] added that the ideal dose is 3 g, while in the Cochrane systematic review carried out by Esposito et al. in 2013 [6], it was stated that 2 g of amoxicillin also significantly reduces the risk of failure. In the present study, where we focused on dentists who prescribe antibiotics exclusively before surgery, we obtained a mean of 1.2% of the sample that administers 2 g of amoxicillin one hour before the surgical procedure. Arteagoitia et al. [5] carried out their research in Spain and also obtained insufficient data (5.73%) when prescribing 2 g of amoxicillin one hour before surgery or 500 mg of amoxicillin one day before surgery. In a separate study conducted in Spain of 247 professionals, Camps-Font et al. [33] found that only 6.9% prescribed before surgery, with 2 g of amoxicillin one hour before treatment (21.6%) and 750 mg of amoxicillin one day before surgery (21.6%) being the most common prescriptions. In the 2019 work by Rodríguez Sánchez et al. [34], 18.2% of Italian dentists exclusively prescribed preoperative antibiotics, with the prescription of choice being amoxicillin/clavulanic acid one hour before surgery. These percentages are much lower than those obtained by the same author [35] (Rodriguez-Sánchez et al., 2019) when studying the prescription habits of 145 professionals in the Netherlands, where only 32.4% prescribed antibiotics before surgery, with 2 g of amoxicillin one hour before surgery (29.6%) being the prescription of choice. In 2015, Deeb et al. [24] conducted a survey of members of the American College of Oral and Maxillofacial Surgeons; the response rate jumped to 51.6% for pre-surgery prescriptions. However, they did not identify those who only prescribed antibiotics preoperatively, or those who gave prescriptions before and after surgery; therefore, only a relative comparison can be made between studies. However, the prescription of choice was indeed 2 g of amoxicillin one hour before surgery, as in the present study. In the case of Al-Kattan and Al-Shibani’s research on Saudi dentists [1], they stated that 100% prescribed antimicrobials before surgery, with the majority (i.e., 21%) prescribing 1 g of amoxicillin one hour before surgery.

Various systematic reviews state that the postoperative prescription of antibiotics does not appear to be justified [6,8,17,22,32,36,37,38]. In the present work, 19.8% of professionals only prescribed antibiotics in the postoperative period, and it was observed that the majority prescribed a seven-day course of 500 mg of amoxicillin (31.2%), followed by 500 mg of azithromycin over three days (25%). In the study by Rodríguez-Sánchez et al. [34], 8.8% only prescribed in the postoperative period, and, more often than not, 875 mg of amoxicillin along with 125 mg of clavulanic acid was the prescription of choice. These findings are similar to those obtained by Arteagoitia et al. (15.42% of professionals) [5], although the latter typically prescribed 500 mg of amoxicillin over seven days (20%). In the study by Camps-Font [33], 40.5% prescribed 750 mg of amoxicillin over seven days. In Al-Kattan’s research [1], 41.1% prescribed a postoperative treatment, with 500 mg of amoxicillin over three days (45.5%) being the most common prescription, followed by 500 mg of amoxicillin over five days (23.6%). The work by Deeb et al. [24] revealed a significant number of prescribers during this period (71.4%), with 500 mg of amoxicillin over five days being the most common prescription (53%). However, they did not make a distinction between those who only prescribed in the postoperative period and those who also received antimicrobials during the preoperative period.

In the present study, 36 dentists (44.4%) gave prescriptions before and after surgery, the most common being 875 mg of amoxicillin along with 125 mg of clavulanic acid two days before surgery for a total of 7–8 days. When comparing with other works, variable results were obtained. Deeb et al. [24], for example, found that 34% of professionals prescribed similarly, although they did not indicate an antibiotic or dosage of choice. These results are consistent with those obtained by Camps-Font et al. [33], for whom 38.1% of dentists prescribed 750 mg of amoxicillin over seven days as the antibiotic and course of choice. The works by Arteagoitia et al. and Rodríguez-Sánchez et al. [5,34] reported that a sizeable 78.85% and 72% of dentists, respectively, carried out the same process. In the case of Arteagoitia et al. [5], it was indicated that dentists prescribed 875 mg of amoxicillin and 125 mg of clavulanic acid most frequently, to be taken over eight days (starting one day before surgery).

It is important to note that one of the major limitations of the present study is that it relies on the relative representation of the sample size. When the survey was conducted, no official census regarding the number of active dental professionals in the Dominican Republic, independently of its area of expertise, was available. Therefore, extrapolation of the results obtained should be done carefully. Guidelines established by an official representative body would also be appropriate.

## 4. Materials and Methods

The research protocol was approved by Federico Henríquez y Carvajal University’s Ethics Committee (1/02/2015).

This cross-sectional observational study was carried out on dentists in Santo Domingo, the Dominican Republic, who attended a conference organized by Federico Henríquez y Carvajal University in 2015 for teachers’ improvement. These internal days were attended by professors from the University and dentists from Santo Domingo invited by the professors.

After reading a summary of the study and its implications, attendees were asked to sign an informed consent form if they agreed to participate in the present study. They later completed a hard-copy survey comprising two sections: the first section contained demographic data (age, sex, years of experience, etc.), and the second was related to antibiotic use in implantology. They discussed issues related to the prescription of antimicrobials, as well as possible prophylactic measures, along with postsurgical treatment and management of complications in healthy and allergy-free patients. The survey was delivered and collected by one of the researchers in this study.

At the time of the study, there was no official census of the number of dentists working in the Dominican Republic. Therefore, it was also not possible to obtain the number of dentists who perform implant surgeries in Santo Domingo, and, because of that, the present study was based solely on the insights of those attending a teaching session conducted by the university.

As inclusion criteria, it was established that only those surveys of dentists who wanted to participate in the study and placed implants would be accepted. Dentists who did not meet the inclusion criteria, as well as those whose surveys were incomplete when reviewed, were excluded from the study. 

Data were compiled and analyzed using Microsoft Excel 2013 (Microsoft Corporation, Redmond, WA, USA) and SPSS version 24.0 (SPSS Inc., Chicago, IL, USA). 

Numerical analysis was undertaken to determine the current state of the habits of dentists vis-à-vis preoperative and postoperative antibiotics, along with complications in the placement of dental implants. For this purpose, the percentages of the number of responses per prescription were calculated, as well as the averages of professional experience and self-perception of the level of knowledge in antibiotherapy. Open-text responses were presented as a nominal response.

## 5. Conclusions

Within certain limits, this study shows that dentists in Santo Domingo, the Dominican Republic, prescribe antibiotics for implant surgery to varying degrees. Dentists’ self-perception of basic antimicrobial knowledge is substandard and should be reinforced with refresher courses. Bridging the gaps in knowledge on the subject could help to standardize prescription guidelines. 

## Figures and Tables

**Table 1:** Prescription guidelines among dentists in Santo Domingo (Dominican Republic).

Antibiotics Prior to Implant Surgery					
Prescription habit	Sample distribution	*N*	YCE	PKU	PKS
Before surgery	1.2%	1	32	10	10
After surgery	19.8%	16	20.1	7.5	8.1
Before and after surgery	44.4%	36	16.6	6.6	7.8
In certain situations	8.6%	7 *	15	7.1	8.1
Never	25.9%	21	18.4	6.9	7.8

YCE, average number of years of clinical experience; PKU, average perception of knowledge acquired during undergraduate studies; PKS, average perception of knowledge acquired through the reading of scientific literature. * Their reasons for deciding to prescribe antibiotics were: gingival inflammation (*N* = 1), patients with heart disease (*N* = 2), patients with systemic disease (*N* = 2), extensive surgeries (*N* = 1), and surgery requiring the use of membrane and bone grafting (*N* = 1).

**Table 2:** Prescription only after surgery.

3-Day Course			
Antibiotic	Dose	Sample distribution	*N*
Azithromycin	500 mg	25%	*4*
*5-Day Course*			
Antibiotic	Dose	Sample distribution	*N*
Amoxicillin	500 mg	12.5%	*2*
*7-Day Course*			
Antibiotic	Dose	Sample distribution	*N*
Amoxicillin	500 mg	31.2%	*5*
Amoxicillin + clavulanic	875 mg	12.5%	*2*
Clindamycin	300 mg	12.5%	*2*
Clindamycin	600 mg	6.25%	*1*

**Table 3:** Prescription guidelines before and after surgery.

Before Surgery			
IMMEDIATELY BEFORE			
Antibiotic	Dose	Sample distribution	*N*
Amoxicillin	500 mg	2.8%	1.0
Azithromycin	500 mg	5.6%	2.0
Amoxicillin + clavulanic	875 mg	2.8%	1.0
1 HOUR BEFORE			
Antibiotic	Dose	Sample distribution	*N*
Amoxicillin	1000 mg	2.8%	1
Amoxicillin	2000 mg	5.6%	2
Azithromycin	500 mg	5.6%	2
1 DAY BEFORE			
Antibiotic	Dose	Sample distribution	*N*
Amoxicillin	500 mg	5.6%	2.0
Azithromycin	500 mg	13.9%	5.0
Amoxicillin + clavulanic	875 mg	22.2%	8.0
2 DAYS BEFORE			
Antibiotic	Dose	Sample distribution	*N*
Amoxicillin	500 mg	5.6%	2.0
Amoxicillin + clavulanic	875 mg	27.8%	10.0
AFTER SURGERY			
2-DAY COURSE			
Antibiotic	Dose	Sample distribution	*N*
Azithromycin	500 mg	25%	9
3-DAY COURSE			
Antibiotic	Dose	Sample distribution	*N*
Azithromycin	500 mg	2.8%	1
Amoxicillin	500 mg	2.8%	1
5-DAY COURSE			
Antibiotic	Dose	Sample distribution	*N*
Amoxicillin	500 mg	2.8%	1
Amoxicillin + clavulanic	875 mg	27.8%	10
			
7-DAY COURSE			
Antibiotic	Dose	Sample distribution	*N*
Amoxicillin	500 mg	5.6%	2
Amoxicillin + clavulanic	875 mg	33.3%	12

## References

[1] Al-Kattan R., Al-Shibani N. (2019). Current trends in antibiotic prescription behavior among Saudi dentists performing implant surgery: A cross-sectional observational study. J. Investig. Clin. Dent..

[2] Alghamdi H.S., Jansen J.A. (2020). The development and future of dental implants. Dent. Mater. J..

[3] Chappuis V., Avila-Ortiz G., Araújo M.G., Monje A. (2018). Medication-related dental implant failure: Systematic review and meta-analysis. Clin. Oral Implants Res..

[4] Khalil D., Hultin M., Andersson Fred L., Parkbring Olsson N., Lund B. (2015). Antibiotic prescription patterns among Swedish dentists working with dental implant surgery: Adherence to recommendations. Clin. Oral Implants Res..

[5] Arteagoitia I., Rodríguez-Andrés C., Rodríguez-Sánchez F. (2018). Antibiotic prophylaxis habits in dental implant surgery among dentists in spain. A cross-sectional survey. Med. Oral Patol. Oral y Cir. Bucal.

[6] Esposito M., Grusovin M.G., Worthington H.V. (2013). Interventions for replacing missing teeth: Antibiotics at dental implant placement to prevent complications. Cochrane Database Syst. Rev..

[7] Laskin D.M., Dent C.D., Morris H.F., Ochi S., Olson J.W. (2000). The Influence of Preoperative Antibiotics on Success of Endosseous Implants at 36 Months. Ann. Periodontol..

[8] Ahmad N., Saad N. (2012). Ahmad Effects of Antibiotics on Dental Implants: A Review. J. Clin. Med. Res..

[9] Binahmed A., Stoykewych A., Peterson L. (2005). Single preoperative dose versus long-term prophylactic antibiotic regimens in dental implant surgery. Int. J. Oral Maxillofac. Implants.

[10] Camacho-Alonso F., Muñoz-Cámara D., Sánchez-Siles M. (2019). Attitudes of dental implantologists in Spain to prescribing antibiotics, analgesics and anti-inflammatories in healthy patients. Med. Oral Patol. Oral y Cir. Bucal.

[11] Abu-Ta’a M., Quirynen M., Teughels W., Van Steenberghe D. (2008). Asepsis during periodontal surgery involving oral implants and the usefulness of peri-operative antibiotics: A prospective, randomized, controlled clinical trial. J. Clin. Periodontol..

[12] Anitua E., Aguirre J.J., Gorosabel A., Barrio P., Errazquin J.M., Román P., Pla R., Carrete J., de Pedro J., Orive G. (2009). A multicentre placebo-controlled randomised clinical trial of antibiotic prophylaxis for placement of single dental implants. Eur. J. Oral Implantol..

[13] Caiazzo A., Casavecchia P., Barone A., Brugnami F. (2011). A pilot study to determine the effectiveness of different amoxicillin regimens in implant surgery. J. Oral Implantol..

[14] Esposito M., Cannizzaro G., Bozzoli P., Checchi L., Ferri V., Landriani S., Leone M., Todisco M., Torchio C., Testori T. (2010). Effectiveness of prophylactic antibiotics at placement of dental implants: A pragmatic multicentre placebo-controlled randomised clinical trial. Eur. J. Oral Implantol..

[15] Braun R.S., Chambrone L., Khouly I. (2019). Prophylactic antibiotic regimens in dental implant failure: A systematic review and meta-analysis. J. Am. Dent. Assoc..

[16] Dawson-Hahn E.E., Mickan S., Onakpoya I., Roberts N., Kronman M., Butler C.C., Thompson M.J. (2017). Short-course versus long-course oral antibiotic treatment for infections treated in outpatient settings: A review of systematic reviews. Fam. Pract..

[17] Rodríguez Sánchez F., Rodríguez Andrés C., Arteagoitia I. (2018). Which antibiotic regimen prevents implant failure or infection after dental implant surgery? A systematic review and meta-analysis. J. Cranio-Maxillofac. Surg..

[18] Arduino P.G., Tirone F., Schiorlin E., Esposito M. (2015). Single preoperative dose of prophylactic amoxicillin versus a 2-day postoperative course in dental implant surgery: A two-centre randomised controlled trial. Eur. J. Oral Implantol..

[19] Sukumar S., Martin F.E., Hughes T.E., Adler C.J. (2020). Think before you prescribe: How dentistry contributes to antibiotic resistance. Aust. Dent. J..

[20] Haque M., Sartelli M., Haque S.Z. (2019). Dental Infection and Resistance-Global Health Consequences. Dent. J..

[21] Sekhar C.H., Narayanan V., Baig M.F. (2001). Role of antimicrobials in third molar surgery: Prospective, double blind, randomized, placebo-controlled clinical study. Br. J. Oral Maxillofac. Surg..

[22] Park J., Tennant M., Walsh L.J., Kruger E. (2018). Is there a consensus on antibiotic usage for dental implant placement in healthy patients?. Aust. Dent. J..

[23] Pyysalo M., Helminen M., Antalainen A.K., Sándor G.K., Wolff J. (2014). Antibiotic prophylaxis patterns of Finnish dentists performing dental implant surgery. Acta Odontol. Scand..

[24] Deeb G.R., Soung G.Y., Best A.M., Laskin D.M. (2015). Antibiotic prescribing habits of oral and maxillofacial surgeons in conjunction with routine dental implant placement. J. Oral Maxillofac. Surg..

[25] Abukaraky A.E., Afifeh K., Khatib A.A., Khdairi N.O., Habarneh H.M., Ahmad W.K., Hamdan A.A., Sawair F.A. (2011). Antibiotics prescribing practices in oral implantology among jordanian dentists. A cross sectional, observational study. BMC Res. Notes.

[26] Ireland R.S., Palmer N.O., Lindenmeyer A., Mills N. (2012). An investigation of antibiotic prophylaxis in implant practice in the UK. Br. Dent. J..

[27] Gill A.S., Morrissey H., Rahman A. (2018). A systematic review and meta-analysis evaluating antibiotic prophylaxis in dental implants and extraction procedures. Medicina.

[28] Khouly I., Braun R.S., Chambrone L. (2019). Antibiotic prophylaxis may not be indicated for prevention of dental implant infections in healthy patients. A systematic review and meta-analysis. Clin. Oral Investig..

[29] Prestinaci F., Pezzotti P., Pantosti A. (2015). Antimicrobial resistance: A global multifaceted phenomenon. Pathog. Glob. Health.

[30] Pisarnturakit P.P., Sooampon S., Sooampon S. (2020). Managing knowledge for health care quality: An investigation of rational antibiotic use among Thai dentists. Int. J. Health Plan. Manag..

[31] El-Kholey K.E., Wali O., Elkomy A., Almozayen A. (2018). Pattern of antibiotic prescription for oral implant treatment among dentists in Saudi Arabia. Implant Dent..

[32] Romandini M., De Tullio I., Congedi F., Kalemaj Z., D’Ambrosio M., Laforí A., Quaranta C., Buti J., Perfetti G. (2019). Antibiotic prophylaxis at dental implant placement: Which is the best protocol? A systematic review and network meta-analysis. J. Clin. Periodontol..

[33] Camps-Font O., Viaplana-Gutiérrez M., Mir-Mari J., Figueiredo R., Gay-Escoda C., Valmaseda-Castellón E. (2018). Antibiotic prescription for the prevention and treatment of postoperative complications after routine dental implant placement. A cross-sectional study performed in Spain. J. Clin. Exp. Dent..

[34] Rodríguez Sánchez F., Arteagoitia I., Rodríguez Andrés C., Caiazzo A. (2019). Antibiotic prophylaxis habits in oral implant surgery among dentists in Italy: A cross-sectional survey. BMC Oral Health.

[35] Sánchez F.R., Arteagoitia I., Andrés C.R., Bruers J. (2019). Antibiotic prophylaxis prescribing habits in oral implant surgery in the Netherlands: A cross-sectional survey. BMC Oral Health.

[36] Ata-Ali J., Ata-Ali F., Ata-Ali F. (2014). Do antibiotics decrease implant failure and postoperative infections? A systematic review and meta-analysis. Int. J. Oral Maxillofac. Surg..

[37] Chrcanovic B.R., Albrektsson T., Wennerberg A. (2014). Prophylactic antibiotic regimen and dental implant failure: A meta-analysis. J. Oral Rehabil..

[38] Lund B., Hultin M., Tranæus S., Naimi-Akbar A., Klinge B. (2015). Complex systematic review—Perioperative antibiotics in conjunction with dental implant placement. Clin. Oral Implants Res..

